# Quality issues with malaria rapid diagnostic test accessories and buffer packaging: findings from a 5-country private sector project in Africa

**DOI:** 10.1186/s12936-017-1820-1

**Published:** 2017-04-20

**Authors:** Steven A. Harvey, Sandra Incardona, Nina Martin, Cristina Lussiana, Elizabeth Streat, Stephanie Dolan, Nora Champouillon, Daniel J. Kyabayinze, Robert Mugerwa, Grace Nakanwagi, Nancy Njoki, Ratsimandisa Rova, Jane Cunningham

**Affiliations:** 1 Department of International Health, Johns Hopkins Bloomberg School of Public Health, 615 N. Wolfe Street, Baltimore, MD 21205 USA; 2 FIND (Foundation for Innovative New Diagnostics), Campus Biotech, Chemin des Mines 9, 1202 Geneva, Switzerland; 3 Population Services International, 1120 19th Street, NW, Suite 600, Washington, DC 20036 USA; 4 Malaria Consortium, Plot 25 Upper Naguru East Road, P.O. Box 8045, Kampala, Uganda; 5 World Health Organization, Global Malaria Programme, 20, Avenue Appia, 1211 Geneva 27, Switzerland

## Abstract

**Background:**

Use of antigen-detecting malaria rapid diagnostic tests (RDTs) has increased exponentially over the last decade. WHO’s Global Malaria Programme, FIND, and other collaborators have established a quality assurance scheme to guide product selection, lot verification, transport, storage, and training procedures. Recent concerns over the quality of buffer packaging and test accessories suggest a need to include these items in product assessments. This paper describes quality problems with buffer and accessories encountered in a project promoting private sector RDT use in five African countries and suggests steps to avoid or more rapidly identify and resolve such problems.

**Methods:**

Private provider complaints about RDT buffer vials and kit accessories were collected during supervisory visits, and a standard assessment process was developed. Using 100 tests drawn from six different lots produced by two manufacturers, lab technicians visually assessed alcohol swab packaging, blood transfer device (BTD) usability, and buffer appearance, then calculated mean blood volume from 10 BTD transfers and mean buffer volume from 10 individual buffer vials. WHO guided complaint reporting and follow-up with manufacturers.

**Results:**

Supervisory visits confirmed user reports of dry alcohol swabs, poorly functioning BTDs, and non-uniform volumes of buffer. Lot testing revealed further evidence of quality problems, leading one manufacturer to replace buffer vials and accessories for 40,000 RDTs. In December 2014, WHO issued an Information Notice for Users regarding variable buffer volumes in single-use vials and recommended against procurement of these products until defects were addressed.

**Discussion:**

Though not necessarily comprehensive or generalizable, the findings presented here highlight the need for extending quality assessment to all malaria RDT test kit contents. Defects such as those described in this paper could reduce test accuracy and increase probability of invalid, false positive, or false negative results. Such deficiencies could undermine provider confidence in RDTs, prompting a return to presumptive treatment or reliance on poor quality microscopy. In partial response to this experience, WHO, FIND, and other project partners have developed guidance on documenting, troubleshooting, reporting, and resolving such problems when they occur.

**Electronic supplementary material:**

The online version of this article (doi:10.1186/s12936-017-1820-1) contains supplementary material, which is available to authorized users.

## Background

With the adoption of artemisinin-based combination therapy (ACT) as the first-line treatment for falciparum malaria in the early 2000s, the World Health Organization (WHO) recommended parasite-based diagnosis first for older children and adults, then for all suspected cases of malaria regardless of patient age [1]. This recommendation led to greatly expanded use of antigen-detecting malaria rapid diagnostic tests (RDTs) worldwide. Total annual RDT sales grew from 46 million in 2008 to 314 million in 2014 [2]. To guide selection of quality RDTs in the face of multiple products on the market, variable reports of test performance, and weak national regulatory systems, WHO, the Foundation for Innovative New Diagnostics (FIND), and other collaborators established the WHO Malaria RDT Product Testing Programme in 2008 at the US Centers for Disease Control and Prevention (CDC) [3]. By December 2015, the programme had completed six rounds of assessment and evaluated 171 products. The programme constitutes the laboratory evaluation component of WHO’s prequalification process for in vitro diagnostics (IVDs) and forms the basis for WHO’s malaria RDT procurement recommendations. Results inform procurement decisions by multilateral and donor agencies, non-governmental organizations (NGOs), ministries of health, and others across the malaria-endemic world [2].

Procuring good quality RDTs, however, does not necessarily guarantee good field performance. Shipping, handling, and storage can affect RDT accuracy as can the training and supervision of health workers in both test preparation and interpretation [4–9]. All have received attention in the literature.

One area that has received less attention is the quality of the buffer and accessories required to conduct RDT testing. The term “accessories” here refers to alcohol swabs, lancets, and blood transfer devices (BTDs). Buffer is referred to as a component. “Accessories” are so described because one brand or style can be substituted for another without compromising test performance. For instance, if tests come packaged with dry alcohol swabs, users can substitute alcohol and cotton or a different alcohol swab. Buffer, in contrast, is manufactured for a specific RDT brand and lot. Substituting buffer from a different brand, lot, or type of test, using water, or using any solution other than the one specifically designed for that lot and test, is contraindicated and could produce incorrect results. For this reason, WHO classifies buffer as a “component”: an integral part of the test. Manufacturers package alcohol swabs, lancets, BTDs, and buffer solution into boxes of 25, 30 or 50 test cassettes, commonly known as ‘hospital packs.’ Alternatively, RDT ‘single packs,’ include a test cassette, accessories and a single-use buffer vial in one envelope. In either case, defective accessories can contribute to anomalous test results. For example, some substandard BTDs can result in collection of too much blood; this can obscure results if the test strip background remains red and test lines illegible. Others can result in collection of too little blood; this can generate false negative results if the blood volume contains insufficient antigen to generate a visible test line. With RDT single packs, single-use vials that contain too little buffer or allow leakage or evaporation can also produce invalid or false negative results. In hospital packs, bottles with insufficient buffer volume may run out before all tests are used. This can lead to tests being wasted or providers substituting water or some other inappropriate solution for buffer. Until recently, however, the WHO-FIND Lot Testing Programme included only limited assessment of buffer and accessories [10].

This paper reports on problems with leakage and stability of single-use buffer vials and quality concerns with accessories encountered in a project promoting RDT use among private sector health care providers in Kenya, Madagascar, Nigeria, Tanzania and Uganda. The paper also describes resulting actions taken by WHO and project partners. The project, “Creating a Private Sector Market for Quality-Assured RDTs in Malaria Endemic Countries,” took place between April 2013 and April 2016. Project details and outcomes will be described elsewhere. Briefly, private providers in the five countries were recruited, trained in malaria diagnosis using RDTs, supplied with an initial stock of test kits, linked with local supply chain partners, and supported by the project’s two implementing partners: Population Services International (PSI) in Kenya, Madagascar, and Tanzania, and Malaria Consortium (MC) in Nigeria and Uganda. WHO, FIND, and the Johns Hopkins Bloomberg School of Public Health (JHSPH) provided support in health worker training, product quality assurance activities, public-sector engagement, and development of relevant guidelines and standard operating procedures (SOPs).

The project’s first year involved working with national regulatory authorities, ministries of health, national malaria control programmes and others to develop or revise guidelines and policies on private sector RDT use. Project partners developed procurement protocols, carried out lot testing on samples drawn from among 500,000 RDTs, recruited and trained an initial cadre of private providers, and engaged with manufacturers, importers, and distributors [11].

## Methods

### Context

Results reported here are based on data from two sources: (1) supervisory and M&E visits to project-based private sector outlets and (2) more extensive assessments carried out off site by FIND and WHO. During 2014, the project trained 801 providers at 682 outlets in the five project countries. Trained providers began offering RDT-based diagnosis in April (Kenya and Madagascar), May (Tanzania), August (Uganda) and September (Nigeria) [12].

### Pre- and post-shipment lot testing

All project-procured RDTs underwent lot testing as per standard protocol prior to shipment from the manufacturer and again after arrival and customs clearance in each project country [13]. Lot testing was carried out at the Research Institute for Tropical Medicine in Manila, Philippines and the Institut Pasteur of Cambodia in Phnom Penh, both WHO-FIND lot testing laboratories [10]. The African Network for Drugs and Diagnostics Innovation (ANDI) Centre of Excellence for Malaria Diagnosis, College of Medicine, University of Lagos, Nigeria, also carried out lot-testing on RDTs used in Nigeria. As part of routine lot testing procedures, major observations—such as partially or completely empty buffer bottles or vials, missing accessories, damaged desiccant sachets, or incorrect package insert sheets—were noted in the comments section of lot testing reports [14].

### Supervisory and M&E visits

Project staff and distributors conducted 478 supervisory visits across the five project countries from June–December 2014. Over the same period, the project M&E team, including representatives from all project partners, visited providers in all five countries. Many providers mentioned problems with buffer vials and test accessories. During routine supervisory visits, Ugandan providers complained of what they perceived to be an unusually large number of negative test results and of vials with insufficient buffer. In response, representatives from the Uganda National Drug Authority (NDA), Malaria Consortium, and FIND visited 65 (31.7%) of the 205 outlets registered with the project in October 2014. To rule out user error, the team observed each provider preparing a test, checking to ensure that the provider (1) followed the preparation instructions correctly, (2) added the correct volume of blood and buffer to the correct test wells, (3) waited the requisite time before interpreting the test results, and (4) correctly interpreted the test outcome. The team then queried each provider about problems with buffer and accessories and recorded any complaints in a log. In a follow-up visit to 95 (46.3%) randomly selected outlets, the team retrieved and evaluated 170 single packs, including test cassettes, single-use buffer vials, BTDs, alcohol swabs, lancets and test instructions [15].

### Qualitative assessment

In January and February 2015, PSI conducted a qualitative study of private provider and client perceptions of RDTs in Tamatave, Madagascar. Study team members interviewed 22 participating providers and 12 adult patients or caregivers of child patients about their experiences using RDTs. While test-kit accessories were not a specific study focus, many providers spontaneously reported problems with lancets, BTDs, alcohol swabs and desiccant packets.

### Accessory assessment during RDT lot testing

#### RDT instructions for use, buffer and accessory review procedures

One partner (MC) requested more extensive assessment of RDT buffer and accessories at the beginning of the project. In response, a standard operating procedure (SOP) was developed and applied to RDTs shipped to Nigeria and Uganda, the two MC project countries (Additional file 1). Qualitative assessments included a review of test instructions, visual inspection of accessories, and observations during lot testing to confirm whether:

1. the instructions clearly described (a) target malaria species, (b) target antigens, (c) correct blood and buffer volumes, (d) proper BTD use, (e) illustrations or text identifying the cassette’s blood and buffer wells, (f) minimum and maximum reading time, and (g) an illustration and text explaining how to interpret test results;
2. BTDs were (a) easy to use (pick-up, transfer and deposit of required blood volume could be done in no more than two attempts) and (b) did not result in blood spillage during transfer;
3. buffer vials appeared intact, buffer colour consistent, and buffer easy to deliver into the test well (dispensing the required volume could be accomplished in a single attempt);
4. alcohol swabs were in sealed undamaged envelopes;
5. desiccant colour indicated that the test kit had not been exposed to excess humidity.

Both buffer vials and BTDs were also assessed quantitatively:

1. Following manufacturer’s instructions, a laboratory technician used a BTD from one RDT test kit to make 10 blood transfers from a lot testing quality control sample. The technician deposited each sample on Whatman 3 M filter paper on a precision balance pre-set to zero and calculated the mean weight of the ten samples. For comparison, ten transfers were then made using a calibrated micropipette set at the manufacturer-specified volume to determine the mean reference weight. The mean blood volume of the 10 BTD transfers was calculated using the reference weight divided by the reference volume as a conversion factor. This was compared with the allowable volume variation when available from the manufacturer.
2. Ten buffer vials selected from individual test kits were used to deposit the manufacturer’s indicated buffer volume in a container on a precision balance pre-set to zero. Mean weight was calculated. The same process was repeated using a calibrated micropipette set at the buffer volume specified by the manufacturer, and calculating the mean reference weight. The buffer volume transferred with vials was calculated using reference weight divided by reference volume, and compared with the allowed volume variation when available from the RDT manufacturer.

Samples from the RDT lots undergoing accessories assessment were provided for either pre-shipment or post-shipment lot testing, with samples drawn by either the RDT manufacturer from the production lot or the project implementer from the shipment pallet in the country port of entry. RDTs were sent to WHO-FIND lot testing laboratories. A total of 100 RDTs were sampled for *Plasmodium falciparum*-only tests, and 150 RDTs for *P. falciparum* plus other species (‘combo’) tests. Shipment and storage were at room temperature throughout the process, and RDTs were assessed within 5 days of receipt at the lot testing laboratories.

## Results

### Supervisory and M&E visits

In Uganda, 13 of 65 outlets visited (20%) reported dry alcohol swabs or single-use buffer vials that were empty, had inconsistent volumes, or were difficult to open or use, resulting in incomplete dispensing of buffer into the cassette. The initial NDA assessment identified five out of 65 providers (7.7%) who reported empty buffer vials. However, the follow-up assessment of 170 kits from 95 providers found that variation in buffer volume fell within acceptable limits based on manufacturer specifications.

**Fig. 1:**
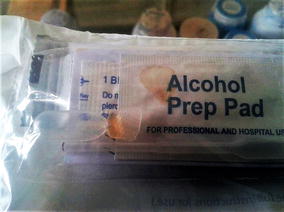
Leakage from buffer vials evidenced by stained packaging (Kenya)

**Fig. 2:**
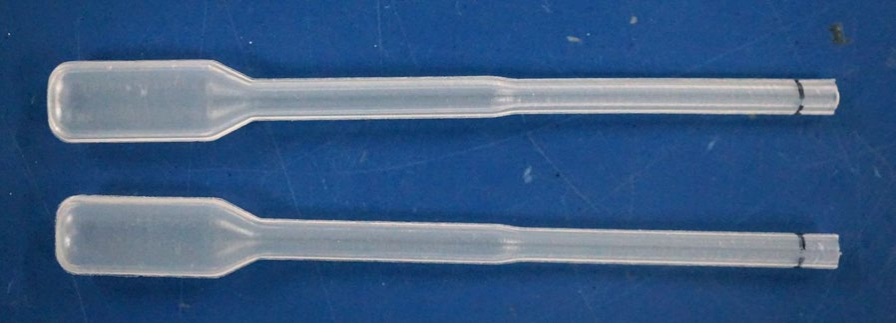
Plastic pipettes designed to collect blood by squeezing a bulb at the* top* (Madagascar)

**Fig. 3:**
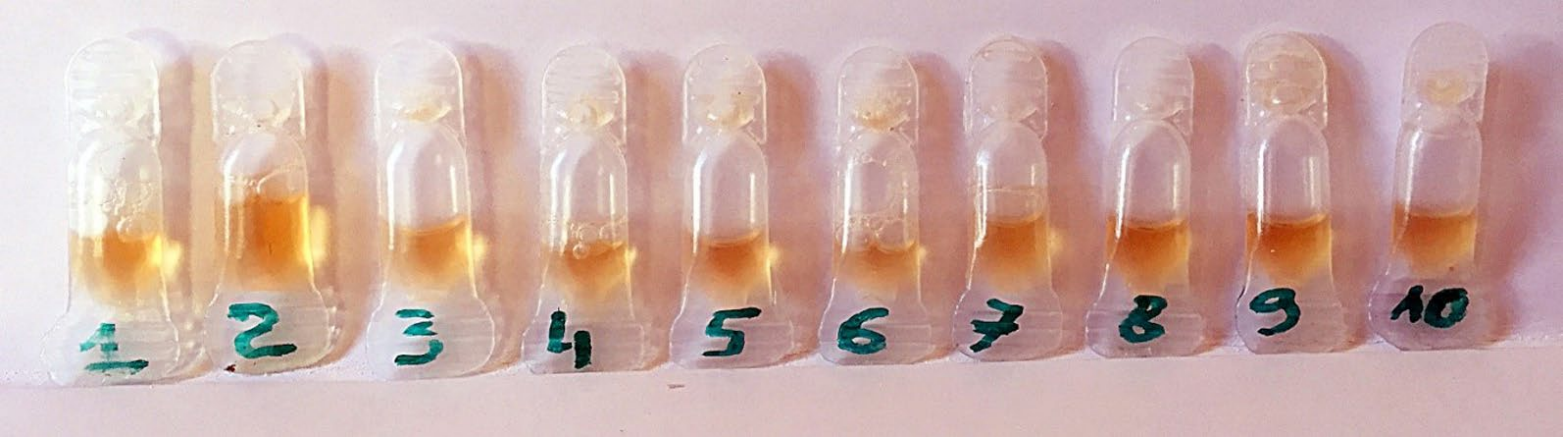
Buffer vials showing different volumes of buffer (Madagascar)

**Fig. 4:**
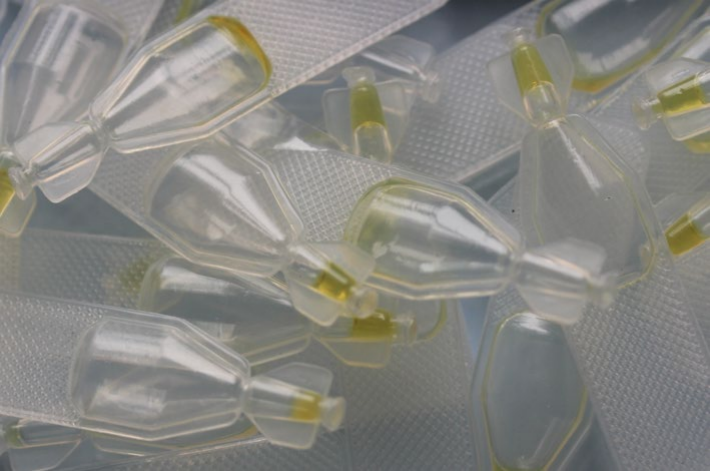
Buffer vials nearly empty with buffer stuck at tip

In Kenya, where accessories assessment was not part of the lot verification done prior to field deployment, quality assurance team visits identified test kits with no BTDs, dry alcohol swabs, and stained packaging indicating buffer vial leakage (Fig. 1). In Madagascar, M&E team members examined accessories collected from a convenience sample of about 20 providers during a September 2014 visit. Almost all alcohol swabs evaluated were completely dry. Test kits contained one-piece eye dropper-like plastic pipettes designed to collect blood by squeezing a bulb at the top (Fig. 2). Stiff plastic made the bulb difficult to compress and blood volume difficult to control. Stiffness also made it difficult to avoid suctioning the blood beyond the pipette tube into the bulb itself from where it could not easily be dispensed into the test cassette. Even when the blood remained in the pipette tube, bulb stiffness hindered delivery into the cassette. Pinholes or cracks in some pipettes also inhibited suction. Plastic flashing at some pipette tips interfered with collecting blood and depositing it into the cassette. Finally, the blood volume indicator line was often not visible. Buffer volume appeared to vary significantly from vial to vial. Some vials appeared nearly or completely empty (Figs. 3, 4).

Half the 22 providers in the early 2015 Madagascar qualitative study mentioned similar findings. Along with dry alcohol swabs, evaporated buffer and defective pipettes, participants reported lancet tips that had detached from their plastic housings or were bent or that broke upon use, and desiccant that had changed colour indicating test exposure to excess humidity.

### Accessory assessment during RDT lot testing

#### Qualitative results

**Fig. 5:**
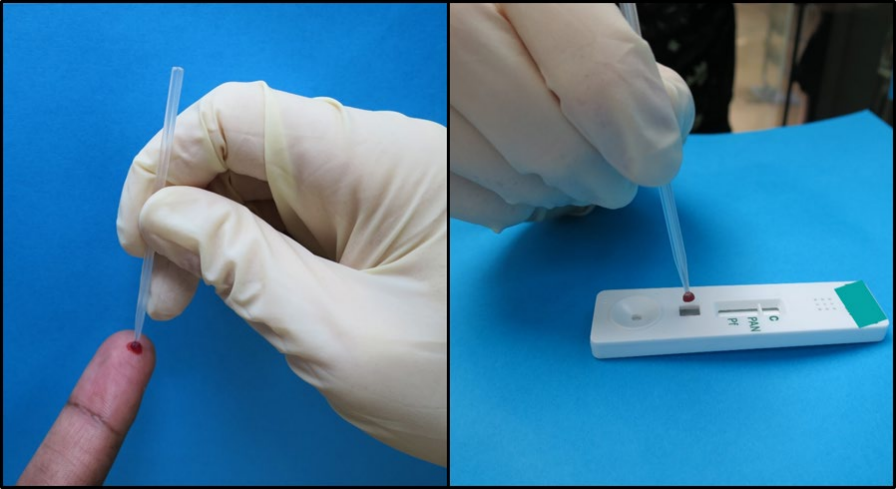
Inverted cup blood collection device (FIND Geneva)
- Buffer vials and alcohol swab envelopes appeared sealed and showed no external evidence of leaking.
- Desiccant packets showed no indication of exposure to humidity.

Lots assessed from Uganda (6) and Nigeria (2) complied with most qualitative criteria:

- Instruction sheets described target malaria species and antigens, blood and buffer volume required, proper BTD and buffer vial use, and minimum and maximum reading times. Sheets included pictures or text indicating correct blood and buffer wells and illustrating how to read and interpret test results.
- The inverted cup BTD (Fig. 5), was rated easy to use and caused no blood spills.

However, for one lot assessed twice during post-shipment testing, once each in Nigeria and Uganda, visual inspection revealed variation in buffer volume and an unusual yellowish colour, possibly indicating evaporation during transport and storage. In both cases, one vial out of 34 randomly opened was completely dry; in others, buffer remained trapped at the tip of the vial and could not be dispensed (Figs. 3, 4).

#### Quantitative results

Table 1 summarizes quantitative BTD results. Variation in mean transferred blood volume ranged from 45.8% below the target 5 µl volume to 9.2% above. However, no manufacturer specified the allowable variation from the 5 µl standard, so it was not possible to determine whether deviations fell within acceptable range.

**Table 1:** Volume of blood collected using pre-packaged blood-collection devices (inverted cup—10 devices per lot tested)

Test lot #	Mean	SD	Min	Max	% variation
1 (Uganda)	2.71	0.56	1.75	3.50	−46
1 (Nigeria)	3.28	0.63	2.27	4.20	−34
2	3.98	0.61	3.07	5.11	−20
3 (Uganda)	3.93	0.49	3.33	4.70	−21
3 (Nigeria)	4.76	0.33	4.39	5.31	−5
4	5.33	0.49	4.29	6.02	7
5	5.46	0.53	4.69	6.12	9
6	5.43	0.62	4.80	6.43	9

All manufacturers specify a 5 μl fingerstick blood sample. % variation is based on the difference between this 5 μl standard and the mean volume in column 2. Lots 1 and 3 were sampled from both Uganda and Nigeria; all other lots were sampled from Nigeria only

Table 2 presents quantitative buffer volume results based on post-shipment lot assessments. While the volume in individual buffer vials sometimes fell outside the manufacturer’s specified allowable variation, the mean volume from five of the seven lot assessments fell within it. Mean volume from the sixth lot, assessed using samples from both Nigeria and Uganda, fell outside acceptable range.

**Table 2:** Volume of buffer (μl) contained in pre-packaged vials (10 vials per lot tested)

Test lot #	Specified volume	Acceptable deviation (μl)	Mean volume	SD	Min–max	Mean volume falls within acceptable deviation?	% variation
1 (Uganda)	70	±10	53.17	18.07	17.78–77.44	No	−24.0%
1 (Nigeria	70	±10	43.32	15.77	21.15–66.88	No	−38.1%
2	130	±39	110.02	9.48	93.48–120.44	Yes	−15.4%
3 (Uganda)	130	±39	109.08	5.48	101.18–118.35	Yes	−16.1
3 (Nigeria)	130	±39	113.06	4.25	107.91–121.07	Yes	−13.0%
4	130	±39	112.12	8.05	95.91–123.11	Yes	−13.8%
5	130	±39	111.34	5.55	103.67–121.53	Yes	−14.4%
6	130	±39	116.13	4.65	108.91–123.98	Yes	−10.7%

Lots 1 and 3 sampled from Uganda and Nigeria; all other lots sampled from Nigeria only. % variation is based on the difference between the manufacturer’s specified volume in column 2 and the mean volume in column 4

## Discussion

This paper’s results reveal several quality issues related to malaria RDT buffer and accessories heretofore not described in the literature. Previous studies have focused primarily on clarity of instructions, package labelling, or desiccant status [16–19]. Some of the issues described here raise more serious concerns than others. Dry alcohol swabs, for instance, may represent only a minor additional cost and inconvenience to providers who must purchase their own alcohol and cotton. Without access to these supplies, however, a provider might wipe the patient’s finger with the dry swab or water or leave it uncleaned, augmenting the risk of infection. Bent, broken or detached lancet tips could pose a risk to both health worker and patient. Some users might procure their own lancets—another additional cost—others might be tempted to substitute a non-sterile or non-disposable device. Poorly functioning BTDs represent an even greater concern. A stiff, difficult to use plastic pipette, manufacturing defects such as pinholes that interfere with suction, or absence of a mark indicating correct volume could result in transferring the wrong blood quantity to the RDT. Excess blood could leave a dark red background in the results window, obscuring test lines. This could cause a health worker to misread a positive result as negative, especially with a low parasite density infection where test lines tend to be faint. Too little blood could also produce false negative results if antigen quantity is insufficient to generate a visible test line. In either case, a malaria-infected patient could go untreated.

Assessment during lot testing showed blood volume variations using the inverted cup, a BTD previously reported as easier to use and more accurate than others [20, 21]. Variations exceeded those in past reports, perhaps because different BTD manufacturers use different designs or plastics. (Note that the Hopkins et al. study was based on 3 transfers from each of 227 health workers; data here are based on 10 transfers with a single BTD by a single technician [21]). The fact that some BTDs show up to 46% volume variation suggests that not all RDT manufacturers perform an adequate quality control of BTDs. Most RDT manufacturers source accessories such as BTDs from other suppliers; under ISO 13485, manufacturers are responsible for verifying the quality of any materials they include in their test kits.

Problems with single-use buffer vials represent another significant concern. An empty vial might tempt an RDT user to substitute water or some other liquid. At least one study has shown that substitution of tap water, distilled water, or saline solution for buffer can all produce false positive results in both *P. falciparum*-only and multi-species RDTs [22]. For vials in which evaporation has left insufficient buffer volume, reagent concentration poses a similar risk. Beyond lots procured for this project, the FIND-WHO RDT lot-testing programme documented buffer evaporation in 145 lots of single-use RDT kits representing 11 different products from three manufacturers between 2014 and 2015 [23]. Anecdotal reports from various countries were also discussed during a meeting of the malaria RDT procurement task force in October 2014. As a result of these various findings, the WHO IVD Prequalification Programme (WHO PQ) issued a notice of concern recommending a halt to procurement and delisting of WHO-prequalified affected products [24]. In parallel, WHO PQ also asked relevant manufacturers to notify those who procured the affected products, investigate the problem, and develop or source alternative, stable, single-use buffer vials. Two affected manufacturers accomplished this using higher density plastic, but sourcing the material and conducting the required stability studies resulted in the product being unavailable for 20 months.

Deficiencies with buffer and accessories could undermine both provider and client confidence in RDTs. It has been widely reported that RDT users doubt negative test results and prescribe antimalarials to RDT-negative patients for fear of leaving an infected but incorrectly diagnosed client untreated [25–28]. Research also shows that both health care providers and patients are reluctant to end a clinical visit without prescribing or receiving some sort of treatment [25–29]. Quality problems with buffer and accessories could reinforce this type of incorrect practice, ultimately leading providers to substitute non-quality assured RDTs or return to poor-quality microscopy or presumptive treatment.

Putting more emphasis on quality assessment of buffer and accessories in WHO laboratory evaluations and WHO PQ manufacturer dossier and site assessments could diminish the likelihood of such problems. This might spur manufacturers to more closely monitor the quality of accessories and RDT components which are often supplied by third parties. FIND, WHO and the other private sector project partners have developed a troubleshooting guide and problems protocol to assist supervisors and end-users in investigating frequently observed anomalous results and errors [30, 31]. The publications offer guidance documenting problems with RDTs, buffer, and accessories and reporting them to NMCPs, national regulatory authorities, and WHO PQ. Timely reporting can help trigger corrective actions such as the previously described WHO PQ notice of concern. Reporting should include investigation to verify that anomalies are due to quality problems rather than end-user errors. The WHO PQ website also accepts problem reports related to malaria and other IVDs [32].

FIND’s expanded assessment has now been updated to include verifying the volume of buffer, the number of accessories and the moisture of alcohol swabs. Because this expanded assessment is highly labour intensive, however, WHO-FIND laboratories can only conduct it by special request on a limited number of lots. A shortened assessment is now included in routine WHO-FIND RDT lot testing, the main difference being that buffer volume uniformity is checked visually rather than measured. Lot testing reports now summarize results and include photos of the accessories. A lot with insufficient buffer for preparing the test will fail the assessment.

The aforementioned interventions would likely reduce buffer- and accessory-related anomalies and provide a mechanism for addressing them at a system level when they occur. They would not, however, respond to the immediate needs of front-line private sector providers who encounter problems with buffer or accessories and need defective items replaced rapidly. Many private providers operate on very narrow financial margins and cannot wait the several months required to resolve an issue through international channels. Similarly, many lack capital to purchase replacement supplies while waiting. During the private sector project, project partners or their distributors could resolve problems more rapidly by intervening directly with international entities and manufacturers. Manufacturers supplying the project generally responded quickly and positively. One manufacturer replaced 40,000 alcohol swabs and BTDs while confirming that individual buffer vial volume, though varied, fell within acceptable limits. Two others also replaced buffer vials after receiving complaints. PSI, MC, and their distributors delivered replacements directly to affected providers.

Outside a project setting, an individual provider facing such problems may lack the wherewithal to contact a manufacturer or regulator directly. An individual provider might likewise have difficulty determining whether a problem was limited or widespread or even whom to contact locally to report one. Guidelines such as those in the aforementioned RDT problems protocol should make it easier for a local or regional distributor to compile provider complaints, pass them on to regulators and manufacturers, and serve as a go-between to ensure that provider concerns are addressed. This could protect private providers from unsustainable losses and reduce provider attrition.

The data reported here have many limitations. Information compiled from the private sector project’s M&E and supervisory visits is based on small non-random samples of providers. Visits included direct qualitative examination of test accessories but did not follow a structured protocol or attempt quantitative measurements (e.g., of blood or buffer volume). The systematic laboratory-based assessment of buffer vials, instructions for use, and accessories, though based on random sampling within lots, included only six lots and two test brands procured for project use in Nigeria and Uganda. Lots for other project countries were procured before the accessories assessment was in place. Further, while the systematic assessment made it possible to quantify the volume of blood transferred to each test, all tests evaluated used the inverted cup BTD, widely considered the most accurate and easiest to use device currently available [21]. Testing with other types of BTD would have provided a more representative picture of the accuracy of devices currently in use. It should also be noted that the assessment is not fully representative of real life since the blood used in testing the BTDs came from a frozen quality control sample, typically less viscous than fresh blood, and was transferred to Whatman filter paper which has a different structure and density than the RDT sample pad. Nevertheless, the procedure allows for a standardized approach to assessing BTDs. Systematic assessment enables quantitative measurement of buffer volume, but leaves open the question of whether the lower-than-specified volume observed is due to inadequate quality control in manufacturing, evaporation during storage, or some other cause. To resolve this, manufacturers would need to carry out root cause analysis. During initial accessory assessments, alcohol swabs were not checked for moisture, lancets were not examined, and accessories were not counted to ensure that their numbers were sufficient. Subsequently, assessment procedures have been updated to address some of these shortcomings, and a shortened procedure for accessories assessment is now incorporated into routine RDT lot testing [13].

Taken together, these limitations preclude generalizable conclusions about the extent of quality issues related to buffer and accessories or the effect of such issues on test performance and acceptability. However, quality issues clearly exist and manufacturers must apply good quality control standards to buffer vials and test accessories as well as to the test devices themselves. Both private and public entities that procure RDTs should be aware of these issues and have a system in place to document, troubleshoot, and rapidly resolve them. This should include a mechanism for replacing defective products on a timeline acceptable to front-line providers. Programmes that evaluate rapid test performance should include assessment of buffer and accessories as an integral part of routine quality control procedures.

## Additional note

A key objective of publishing this paper was to highlight the need for more effective quality control of buffer and accessories for all malaria RDTs, something the authors believe merits greater attention than it has received to date. As a result, the authors elected not to name specific brands or products. Manufacturers of all the products described here have either discontinued sale of these products or remedied the problem by sourcing new materials that meet higher quality control standards. Further, since the sample described here was small and non-representative, it is possible that the buffer or accessories packaged with other brands of RDTs suffer from the same or similar defects that have not yet come to light. Singling out one or two manufacturers for specific mention might lead some readers to conclude that both the problem and the recommendations for improved quality control relate to a small number of specific brands or products rather than to an industry-wide issue.

## Additional file

**Additional file 1.** Standard Operation Procedures (SOP) 04.00: Assessment of accessories of individually packaged malaria RDT kits.

## Abbreviations

ACT: artemisinin-based combination therapy
ANDI: African Network for Drugs and Diagnostics Innovation
BTD: blood transfer device
CDC: US Centers for Disease Control and Prevention
FIND: Foundation for Innovative New Diagnostics
IVDs: in-vitro diagnostics
JHSPH: Johns Hopkins Bloomberg School of Public Health
MC: Malaria Consortium
NDA: Uganda National Drug Authority
NGO: non-governmental organization
PSI: Population Services International
RDT: rapid diagnostic test
SOP: standard operating procedure
TDR: WHO Special Programme for Research and Training in Tropical Diseases
WHO: World Health Organization
WHO-PQ: WHO IVD Prequalification Programme
