# Supplementary material for: Quality issues with malaria rapid diagnostic test accessories and buffer packaging: findings from a 5-country private sector project in Africa
Source: Malar J. 2017 Apr 20;16:160. doi: 10.1186/s12936-017-1820-1 (PMC5397682; doi:10.1186/s12936-017-1820-1)
Supplement: Supplementary file 1 — Additional file 1. Standard Operation Procedures (SOP) 04.00: Assessment of accessories of individually packaged malaria RDT kits. [file 12936_2017_1820_MOESM1_ESM.docx]

**SOP 04.00 Assessment of accessories of individually packaged malaria RDT kits**

#### **PURPOSE**

This Standard Operating Procedure (SOP) describes the assessment of accessories contained in individually packaged malaria RDTs, to determine if they are all appropriate and satisfyingly fulfill their role. This is an optional assessment, complementary to the lot testing of test performance (sensitivity) with frozen blood QC samples

#### **SCOPE**

This procedure is to be used in complement to procedures of chapter 2 of the “Methods Manual for laboratory quality control testing of malaria rapid diagnostic tests”, when assessment of the quality of RDT test kit accessories is desired. The SOP is only to be modified with agreement of the Designated FIND or WHO Officer.

#### **PROCEDURE**

The following procedure describes assessment of certain accessories by simple inspection, while others will need to be assessed by performing RDTs. Observations while performing RDTs should therefore be recorded during the process of lot testing with the frozen QC samples according to SOP 2.06. If only the assessment of accessories is carried out (no lot testing), then the observations should be based on performance of at least 5 RDTs.

All observations and results of the following assessment steps are to be recorded on Form 04.01.

###### **A. Product insert sheet**

1. The Product insert sheet should be available in English, and/or in the locally most common language that can be read, written and understood by all health staff in the relevant country.
2. It should clearly state the malaria species and antigen(s) detected by the RDT, and specify which test line detects which antigen, in a picture of the RDT.
3. The procedure for performing the RDT should be explained both in an easily understandable text as well as with pictures visualizing each of the critical steps. The following information should be clearly specified:

- Dispensing the blood: exact volume of blood, how to use the blood transfer device, specify in a picture in which well of the RDT cassette the blood is to be deposited,
- Dispensing the buffer: exact volume and/or exact number of drops of buffer, how to use the buffer ampoules, and specify in a picture in which well of the RDT cassette the buffer is to be deposited,
- Reading time: specify the minimum reading time; eventually specify a maximum reading time not to exceed,
- Test result interpretation: the text should explain, and pictures should show how to interpret test results, with examples of positive test results, for each species in the case of combination tests, as well as an example of a negative test result and of an invalid test result.

###### **B. Blood transfer device**

1. Ease of use and safety:

The following steps must be easy to complete, with “easy” meaning that not more than two attempts are required to complete the step:

- Pick-up of the desired volume of blood
- Transfer of the blood from the fingerprick to the RDT
- Deposit of the full volume of blood

Additionally, no blood spillage should be observed during the blood transfer, supposing that transfer is done without any brisk movements and without touching anything with the device.

1. Blood volume assessment:

- Transfer 10 times the recommended blood volume by using the blood transfer device (BTD) as described in the insert sheet, and deposit on a piece of Whatman 3M filter paper or equivalent on a microbalance already set at zero. Record the weight after each individual transfer, then calculate the mean value, and complete Form 04.02.
- Repeat the same procedure by using a micropipette to transfer the target volume as specified in the product insert sheet. If the target volume is not specified, then it should be obtained from the requester of this assessment (who can obtain this information from the manufacturer).
- Using the obtained mean values, convert the mean weight obtained for the BTD into a mean volume as specified in Form 4.02.

###### **C. Buffer – individual ampoules**

1. Inspect at least ten different buffer ampoules, and verify that the color and transparency of the liquid are consistent and that all ampoules are intact
2. The use of the buffer ampoules should not require more than one attempt to dispense the required buffer volume on a RDT (easy delivery of buffer).
3. Buffer volume assessment:

- Based on instructions described in the product insert sheet, deposit 10 times the recommended buffer volume with the buffer ampoules in a small container on a microbalance set at zero. Record the weight after each individual transfer, then calculate the mean value, and complete Form 04.03.
- Repeat the same procedure by using a micropipette to transfer the target volume as specified in the product insert sheet. If the target volume is not specified, then it should be obtained from the requester of this assessment (who can obtain this information from the manufacturer).
- Using the obtained mean values, convert the mean weight obtained for the BTD into a mean volume as specified in Form 4.03.

###### **D. Other accessories**

1. Alcohol swabs should be in closed, undamaged envelopes.
2. The color of the dessicant should be standard and not indicate any color change due to humidity. If there is no information in the insert sheet regarding the standard color, versus color of humidity-exposed dessicant, then assess this by putting some dessicant in presence of water and note if and what color change occurs. The information about the standard color, versus the color of humidity-exposed dessicant, can also be obtained from the requester of the assessment, who can obtain it from the manufacturer. If color does not change after exposure to water, then note ‘NA’ (not applicable) in the assessment form.

###### **E. Reporting**

1. Complete Form 4.01 with all observations.
2. Interpretation of the blood and buffer volume assessments:

- The target (reference) volumes for both the blood and the buffer transfers are usually specified in the product insert sheet (in microliters). If not, then they should be obtained from the requester of this assessment (who can obtain this information from the manufacturer).
- The allowed volume variations are usually not specified in the product insert sheet. Unless it is specified, obtain the allowed variations for blood and buffer transfers from the requester of this assessment (who can obtain this information from the manufacturer).
- Calculate the % of variation between the mean volume obtained from the 10 transfers with the device (BTD or buffer ampoule) and the target volume specified by the manufacturer, and complete Form 4.01.
- The mean volume transferred with the accessory (BTD or buffer ampoule) (**Vol B**) should be within the specified variation (X%) of the reference volume specified by the manufacturer (**Vol R**), i.e.

Vol R – (X * Vol R)/100 < **Vol B** < Vol R + (X * Vol R)/100

- If the information about the allowed volume variation cannot be obtained, then note “unknown” in the relevant fields of Forms 4.01-4.03 that cannot be determined.

1. Complete the final appreciation fields of Form 4.01, obtain signature from the head of laboratory, and send the full report (Forms 4.01 to 4.03) to the recipients specified in the Lot Testing Request form.
2. It is the responsibility of the requester to determine what assessment results are considered acceptable or are subject to lot rejection. These acceptance/rejection criteria should be discussed with the manufacturer and ideally be integrated in the procurement contract. The final decision about acceptance or rejection is to be taken by the requester. The FIND and WHO Project managers can assist in discussions with the manufacturer if needed, but only for providing further explanations on the assessment procedure and results interpretation. FIND and WHO don’t have any role in arbitration of eventual conflicts.

## Form 04.01: Assessment of RDT kit accessories

Date: ___/___/_______ (dd/mm/yyyy) Technician: _________________________

**RDT KIT**

| RDT (name/brand) | Manufacturer | Catalog number | Lot Number | Expiry date |
| --- | --- | --- | --- | --- |
|  |  |  |  |  |

**RESULTS** (check the field of the relevant answers)

| Assessed item | Result | Comment |
| --- | --- | --- |
| **Product Insert sheet** | | |
| In English or other common language? | YES ( ) No ( ) NA ( ) |  |
| Target malaria species specified? * | YES ( ) No ( ) NA ( ) |  |
| Target antigens specified for each test line? | YES ( ) No ( ) NA ( ) |  |
| Picture showing target antigen and/or species for each test line? | YES ( ) No ( ) NA ( ) |  |
| Volume of blood specified? | YES ( ) No ( ) NA ( ) |  |
| Use of blood transfer device explained? | YES ( ) No ( ) NA ( ) |  |
| Picture/text explaining appropriate well for blood? | YES ( ) No ( ) NA ( ) |  |
| Volume of buffer specified (number of drops or volume)? | YES ( ) No ( ) NA ( ) |  |
| Use of individual buffer ampoules explained? | YES ( ) No ( ) NA ( ) |  |
| Picture/text explaining appropriate well for buffer? | YES ( ) No ( ) NA ( ) |  |
| Minimum reading time specified? | YES ( ) No ( ) NA ( ) |  |
| Maximum reading time specified? | YES ( ) No ( ) NA ( ) |  |
| Text AND picture explaining test results interpretation for: | | |
| - Each of the detected species (positive) | YES ( ) No ( ) NA ( ) |  |
| - Negative result | YES ( ) No ( ) NA ( ) |  |
| - Invalid test result | YES ( ) No ( ) NA ( ) |  |
| **Blood transfer devices (BTD)** | | |
| Easy pick-up of blood? | YES ( ) No ( ) NA ( ) |  |
| Easy transfer of blood? | YES ( ) No ( ) NA ( ) |  |
| Easy deposit of blood? | YES ( ) No ( ) NA ( ) |  |
| No risk of spillage during transfer? | YES ( ) No ( ) NA ( ) |  |
| Mean volume within the allowed variation of the specified target volume | YES ( ) No ( ) NA ( )  Unknown ( ) |  |
| **Buffer ampoules** | | |
| Buffer ampoules appear to be intact | YES ( ) No ( ) NA ( ) |  |
| Aspect of buffer appears to be consistent | YES ( ) No ( ) NA ( ) |  |
| Easy delivery of buffer into well? | YES ( ) No ( ) NA ( ) |  |
| Mean volume within the allowed variation of the specified target volume | YES ( ) No ( ) NA ( )  Unknown ( ) |  |
| **Alcool swabs** in intact envelopes | YES ( ) No ( ) NA ( ) |  |
| **Dessicant** does not indicate exposure to humidity | YES ( ) No ( ) NA ( ) |  |

**FINAL APPRECIATION:**

**All assessment items are successfully fulfilled: ( )**

**Some assessment items are not fulfilled: ( )**

**Number of unfulfilled assessment items: ___ out of 27**

**Comments: _____________________________________________________________**

**_______________________________________________________________________**

**_______________________________________________________________________**

***Signed:***

**Technician Laboratory Head**

## Form 04.02: Assessment of blood transfer devices (volume)

Date: ___/___/_______ (dd/mm/yyyy) Technician: __________________________

**RDT KIT**

| RDT (name/brand) | Manufacturer | Catalog number | Lot Number | Expiry date |
| --- | --- | --- | --- | --- |
|  |  |  |  |  |

Type of blood transfer device (loop/straw/squeezable pipette/capillary/inverted cup/others):

**_________________________**

**RESULTS**

**Blood transfer device Micropipette**

| Deposit n° | Weight (g) |  | Deposit n° | Weight (g) |
| --- | --- | --- | --- | --- |
| 1 |  |  | 1 |  |
| 2 |  |  | 2 |  |
| 3 |  |  | 3 |  |
| 4 |  |  | 4 |  |
| 5 |  |  | 5 |  |
| 6 |  |  | 6 |  |
| 7 |  |  | 7 |  |
| 8 |  |  | 8 |  |
| 9 |  |  | 9 |  |
| 10 |  |  | 10 |  |
| **Mean weight B** |  |  | **Mean weight R** |  |
| \| **Reference (Vol R) in ul** \|  \| \| --- \| --- \|   **Reference volume specified by the manufacturer:**  **Conversion factor of weight into volume = Mean weight R / Reference volume R = ­­­­­­­­­­­­­­­­­­­­­­­­­­­_______**   \| **Mean (Vol B) in ul** \|  \| \| --- \| --- \|   **Mean Volume B = Mean weight B / Conversion factor:** | | | | |

| **Variation (X) in %** |  |
| --- | --- |

**Allowed variation specified by the manufacturer:**

**Mean volume transferred with the BTD within allowed variation of the specified reference volume:**

Vol R – (X * Vol R)/100 < **Vol B** < VolR + (X * Vol R)/100 ?

**YES ( ) NO ( ) UNKNOWN ( )**

## Form 04.03: Assessment of buffer ampoules (volume)

Date: ___/___/_______ (dd/mm/yyyy) Technician: __________________________

**RDT KIT**

| RDT (name/brand) | Manufacturer | Catalog number | Lot Number | Expiry date |
| --- | --- | --- | --- | --- |
|  |  |  |  |  |

**RESULTS**

Buffer ampoules Micropipette

| Deposit n° | Weight (g) |  | Deposit n° | Weight (g) |
| --- | --- | --- | --- | --- |
| 1 |  |  | 1 |  |
| 2 |  |  | 2 |  |
| 3 |  |  | 3 |  |
| 4 |  |  | 4 |  |
| 5 |  |  | 5 |  |
| 6 |  |  | 6 |  |
| 7 |  |  | 7 |  |
| 8 |  |  | 8 |  |
| 9 |  |  | 9 |  |
| 10 |  |  | 10 |  |
| **Mean weight B** |  |  | **Mean weight R** |  |
| \| **Reference (Vol R) in ul** \|  \| \| --- \| --- \|   **Reference volume specified by the manufacturer:**  **Conversion factor of weight into volume = Mean weight R / Reference volume R = ­­­­­­­­­­­­­­­­­­­­­­­­­­­_______**   \| **Mean (Vol B) in ul** \|  \| \| --- \| --- \|   **Mean Volume B = Mean weight B / Conversion factor:** | | | | |

| **Variation (X) in %** |  |
| --- | --- |

**Allowed variation specified by the manufacturer:**

**Mean volume transferred with the BTD within allowed variation of the specified reference volume:**

Vol R – (X * Vol R)/100 < **Vol B** < VolR + (X * Vol R)/100 ?

**YES ( ) NO ( )**
